# Impacting employees’ and managers’ mental health skills using a workplace-adapted mindfulness-based intervention

**DOI:** 10.3389/fpsyg.2022.1020454

**Published:** 2022-12-06

**Authors:** Emilie Hasager Bonde, Eva Gemzøe Mikkelsen, Lone Overby Fjorback, Lise Juul

**Affiliations:** ^1^Department of Clinical Medicine, Danish Center for Mindfulness, Aarhus University, Aarhus, Denmark; ^2^Department of Psychology, University of Southern Denmark, Odense, Denmark

**Keywords:** mental health, mindfulness, workplace, health promotion and prevention, qualitative methods

## Abstract

**Background:**

During the past decades, the mental health of the population has been declining. Mindfulness-based stress reduction (MBSR) has been found effective in enhancing well-being along with reducing perceived stress and symptoms of anxiety and depression. Mindfulness-based interventions (MBIs) in the workplace have shown promising results relating to the mental health of employees and managers. However, the research field of organizational-level MBIs being offered to entire companies is still nascent. Practicing mindfulness may affect skills related to good mental health. Thus, the objective of this study was to investigate the impact of an organizational-level MBI on the mental health skills of employees and managers.

**Methods:**

This qualitative study was part of a quasi-experimental multi-method study. Four small and medium-sized private enterprises with a total of 368 employees and managers were included. The intervention contained: 1. An obligatory introductory session on mental health and mindfulness, 2. Voluntary participation in a 10-week live online workplace-adapted MBSR course, and 3. A workshop for selected employee representatives and managers on further implementation of mindfulness in the organization. A total of 27 focus group interviews including 76 respondents were conducted pre- and post-intervention. Verbatim transcription was performed. Data was analyzed using inductive qualitative content analysis.

**Results:**

Through analysis, four pre-intervention categories emerged: 1. Bodily sensations and awareness in stressful situations, 2. Reactive and passive behavior during stressful situations, 3. Differences in perception as a stressor, 4. Self-criticism and low ability to practice self-care. Six post-intervention categories were identified: 1. Enhanced ability to be aware in the present moment, 2. Increased acknowledgement of how others may view things differently from oneself, 3. Increased kindness to oneself and being able to practice self-care, 4. Moving from reactive to responsive behavior in stressful situations, 5. Mindfulness as an accelerator for an ongoing personal process and 6. Practicing mindfulness – setting time aside or being mindful in everyday life.

**Conclusion:**

This study indicates that it is possible to enhance employees’ and managers’ mental health skills using an organizational-level MBI. Enhanced awareness in the present moment transcended through post-intervention categories, facilitating increased self-kindness and responsive behavior in stressful situations.

## Introduction

During the past decades, the mental health of the European population has been continuously declining (WHO, 2018a). A process which was reinforced by the Covid-19 pandemic. As such, the prevalence of anxiety and symptoms of depression amongst adults in OECD countries has risen dramatically from pre-pandemic 2019 to 2021 (OECD, 2021). In Denmark, the percentage of the population with a low mental health score measured on SF-12 has increased from 13.2% in 2017 to 17.4% in 2021 (Jensen et al., 2022). Moreover, a similar increase in percentage is evident in Danes experiencing a perceived high stress level (Jensen et al., 2022). However, mental health is more than the absence of psychological distress. Indeed, it is defined as: “… *a state of well-being in which an individual realizes his or her own abilities, can cope with the normal stresses of life, can work productively and is able to make a contribution to his or her community*” (WHO, 2018b). Hence, more positive elements such as realization of abilities and capabilities to cope with stress are fundamentals for having good mental health. Consequently, and as stressed by the Covid-19 pandemic, there is a need for implementing mental health promoting and preventing interventions to increase the population’s mental health.

To identify which mental health promoting and preventing interventions to utilize, knowledge of causes related to poor mental health must be obtained. According to the World Health Organization’s (WHO) World mental health report (WHO, 2022), individuals’ mental health depends on multiple factors within the domains of individual factors, family and community factors and structural factors (WHO, 2022). Therefore, interventions working to promote protective factors within the three domains are needed. Protective factors for individuals’ mental health within the individual domain is, i.e., “*sense of self-worth and mastery*” and “*social and emotional skills*” (WHO, 2022). In this study, acquired or intrinsic skills serving as protective factors to one’s mental health are referred to as *mental health skills*. These may be skills such as regulating emotions, self-esteem and capability to engage in interpersonal relations (WHO, 2012).

In WHO’s World mental health report (WHO, 2022), mindfulness-based interventions (MBIs) are mentioned as examples of evidence-based psychosocial interventions that have been found effective in improving mental health (WHO, 2022). Being mindful enables individuals to be aware in the present moment with a kind and curious attitude. Mindfulness is defined as *“… the awareness arising through paying attention on purpose in the present moment, non-judgmentally, in the service of self-understanding, wisdom, and compassion*” (Kabat-Zinn, 2018). Being aware in the present moment allows one to actually experience that moment. It also allows one to notice bodily sensations, mood, thoughts and feelings; A knowledge, which is very helpful in order to be able to respond to life instead of reacting automatically (Fjorback, 2015). In a study by Killingsworth and Gilbert (2010), the authors investigated how not being mentally present in what you are doing affects self-reported happiness (Killingsworth and Gilbert, 2010). Using an App-based approach, they measured individuals’ happiness and the degree to which they were aware in the present moment in real-time. The authors found that, on average, we humans are not mentally present in what we are doing 46.9% of the time, and that participants were happiest when they were mentally present in what they were doing (Killingsworth and Gilbert, 2010). Hence, according to this study, people are less happy when they are not mentally present in the moment – regardless of where to their thoughts might wander (Killingsworth and Gilbert, 2010). Moreover, practicing mindfulness often involves focusing the attention on bodily sensations. Being aware of bodily sensations is proposed to be fundamental in for example self-regulation (Treves et al., 2019).

Mindfulness-based stress reduction (MBSR) is an 8-week programme designed to assist participants in developing abilities to be consciously aware in the present with kindness toward oneself and the surroundings. Furthermore, the curriculum-based programme entails practices focussing on becoming aware of reactions during stress. The programme is delivered by a trained MBSR teacher in a group format with weekly sessions of 2.5 h and a 7-h silent retreat day (Santorelli, 2014; Brown Mindfulness Center, 2020).

Previous research on MBSR has been conducted across study populations including both clinical and non-clinical populations. Furthermore, MBSR has been studied in various settings such as in schools, hospitals and workplaces (De Vibe et al., 2017). Across study populations and settings, MBSR has been found effective in improving mental health among adults (De Vibe et al., 2017). Furthermore, participation in MBSR enhanced coping and empathy (De Vibe et al., 2017), both of which are factors relating to social and emotional skills put forward by the WHO as protective factors of mental health (WHO, 2022). Hence, MBSR is a universal intervention that can be delivered to both clinical and non-clinical populations demonstrating favourable effects on participants’ mental health and protective mental health skills. Moreover, a recent systematic review and meta-analysis found MBIs to be some of the most effective in improving mental well-being compared to other mental health interventions (van Agteren et al., 2021).

According to McHenry (2012), mental health promoting interventions should be implemented in settings where people live their lives, i.e., workplaces (McHenry, 2012). Therefore, there is an incentive to implement mental health promoting and preventive interventions in workplace settings. The research area of implementing MBIs in workplaces is steadily developing with the majority of previous research of MBIs having been conducted within the public sector (Janssen et al., 2018). A systematic review and meta-analysis of randomized controlled trials of MBIs in the workplace showed these interventions to be effective in improving mental health (Vonderlin et al., 2020). Online and App-based MBIs have also demonstrated positive effects regarding stress reduction, mindfulness and concentration (Aikens et al., 2014; Nadler et al., 2020; Axelsen et al., 2022). However, most of these interventions targeted individuals within workplace settings.

According to the Medical Research Council, interventions can effectively target entire organizations to facilitate system change (Skivington et al., 2021). When intervening at an organizational level, a population-based strategy may be utilized. By using a population-based strategy, interventions are provided to the entire risk-spectrum of a population, including low, moderate and high-risk individuals (Rose et al., 2008). Such a risk-spectrum might represent a spectrum ranging from high to low degree of mental health. Hence, when using a population-based strategy, the interventions do not focus on selected high-risk individuals but instead target entire populations (Rose et al., 2008), such as organizations, i.e., workplaces. Utilizing a population-based strategy may prevent individuals at low and moderate risk of, i.e., poor mental health from transcending to the high-risk population, thereby preventing poor mental health in a wider population. In a study by Kersemaekers et al. (2018), they found an organizational-level workplace-adapted MBI effective in reducing perceived stress and enhancing well-being and mindfulness (Kersemaekers et al., 2018). Moreover, they also demonstrated improvements in the organizational climate (Kersemaekers et al., 2018). By intervening at an organizational level, the intervention is offered to the entire staff with the hopes of contributing to the creation of healthier work environments. Additionally, the impact on leader capabilities of this workplace-adapted MBI was investigated in a qualitative study by Rupprecht et al. (2019). In that study, managers showed enhanced abilities in both self-management and management of employees (Rupprecht et al., 2019). However, knowledge of how a workplace-adapted MBI impacts mental health skills among employees and managers is lacking. To gain insights into how employees and managers express the impact of a workplace-adapted MBI on their mental health skills, there is a need for further qualitative research.

The results of previous research may be dependent on context and generalization to other settings and/or study populations might not be feasible (Galante et al., 2021; Skivington et al., 2021). Therefore, there is a need for investigating how an organization-level intervention aimed at implementing a workplace-MBI to as many employees and managers as possible can impact mental health skills.

Accordingly, the main objective of this qualitative study was to gain an understanding of how a workplace-MBI including a workplace-adapted MBSR course impacts the expressed mental health skills of employees and managers employed in small and medium-sized enterprises (SMEs). Secondary, this understanding will generate insights into the feasibility of implementing MBIs in private workplaces and of using workplaces as mental health promoting settings.

## Materials and methods

### Design

The present study presents qualitative results from a quasi-experimental, multi-method trial which main objective was to support the creation of healthy work environments using a workplace-adapted MBI. The main trial was registered with the Danish Data Protection Agency (2016–051-000001/1715). The present qualitative study however focuses on how mental health skills of employees and managers are impacted by a workplace-adapted MBI. The trial enrolled four SMEs with 10–249 employees and managers with the entire company or divisions thereof based in Denmark. The four SMEs represented businesses within media (Company 1), restaurants (Company 2), production (Company 3) and health-IT (Company 4). Company 4 was an international SME with employees working from offices across the globe. The remaining three companies were based in Denmark.

### Participants and recruitment

To be eligible for inclusion in the study, companies were required to be privately owned and have a total of 10–249 employed at enrolment. Furthermore, top management at the companies had to give permission for the employees to spend working hours participating in the intervention.

Companies were recruited *via* multiple channels including direct contact to relevant companies, digital newsletters from business organizations within IT and production businesses, social media post on Twitter, Facebook and LinkedIn as well as posts on the Danish Center for Mindfulness’ webpage. Recruitment was performed between January 2020 and October 2020.

Upon a company’s expressed interest in participation, a preliminary meeting with selected managers hosted by the research project leader, LJ, and an MBSR teacher took place. At this meeting, managers were informed that signing up for the project would require them to enable all employees and managers to participate in an obligatory two-hour information session during working hours. Furthermore, the managers were informed that the intervention were to be an organizational-level intervention. Hence, the intervention was not to be offered only to selected groups of employees and/or managers. The aim was to support a positive mental health environment in the organisation. During the meeting, the research project leader (LJ) emphasized that to participate, employees and managers had to be able to attend a live online 10-weeks workplace-adapted MBSR course either during working hours or with monetary payment for time spent participating during leisure time. After committing to these conditions, a formal contract of participation was signed by a company representative, typically by a person in the top management.

### Intervention

In all companies, the intervention consisted of three components: obligatory participation in a two-hour information-session on mental health and mindfulness for all employees and managers, a live online 10-weeks workplace-adapted MBSR course for self-selecting employees and managers, and lastly a workshop for selected employee representatives and managers on the subsequent implementation of mindfulness in the company (Table 1).

**Table 1:** Intervention components and content.

Component	Content	Delivered by	Participants
Information session	A two-hour lecture concerning: What is mental health? The bodily stress response The possibility to train one’s mental health Mindfulness as a way of training one’s mental health Previous research on MBSR Two guided mindfulness exercises Information about the possibility to participate in a 10-weeks workplace-adapted MBSR course	A certified MBSR teacher	All employees and managers
10-weeks live online workplace-adapted MBSR course	A systematically workplace-adapted 10-week MBSR course including: Same themes as the original MBSR programme Experience-based approach was utilized, engaging participants in mindfulness practices Horizontal inquiry of direct experiences during mindfulness practices Delivered live online *via* Zoom Invitation to practice a minimum of 3 times a week for 15 min	A certified MBSR teacher	Self-selected employees and managers
Implementation workshop	A two-hour workshop focusing on further implementation of mindfulness in the participating companies consisting of: Discussions in groups and plenary about the wish for and possible barriers to further implementation of mindfulness Presentations of ideas immerging from these discussions Facilitators presenting possible ways to further implementation Creating an action plan for further implementation	An organizational psychologist, a certified MBSR teacher and an observant	Selected employee representatives and managers

Participation in a two-hour information-session was obligatory so to ensure that all employees and managers got the same information. The 10-weeks workplace-adapted MBSR course was systematically adapted from the original MBSR curriculum. Adaptation of the original MBSR programme was conducted with caution of what elements of the intervention could be varied to enhance intervention-context-fit, and which intervention elements were essential to ensure reliability of the programme theory (Crane et al., 2017). This workplace-adapted MBSR course followed the original MBSR curriculum with all weekly themes represented along the 10 weeks. Furthermore, a trained MBSR teacher delivered the course live online *via* Zoom in groups of 5–22 employees and/or managers. Through the course, the MBSR teachers utilized an experience-based approach, engaging participants in mindfulness practices followed by horizontal inquiry of direct experiences during the practices (Crane et al., 2017). During the 10-weeks workplace-adapted MBSR courses, LF supervised all MBSR teachers delivering one or more courses in the participating companies in weekly 1.5-h sessions (see Table 2).

**Table 2:** Differences between the original and workplace-adapted MBSR programme.

	Workplace-adapted 10-weeks MBSR course	Original MBSR course
Length of programme	10 weeks	8 weeks
Length of sessions	1.5 h	2.5 h
Silent retreat session	Included as one of the 10 sessions. Duration: 1.5 h	Added as a 9th session within the 8-week programme. Duration: 7 h

### Respondents

Sampling of respondents for the focus group interviews were conducted using the Matrix sampling method (Campbell et al., 2020). This purposive sampling method allowed managers to select employees that represented positive, negative and neutral attitudes towards mindfulness pre-intervention. Post-intervention, the Matrix sampling method was applied to allow MBSR teachers to recommend respondents that had participated in their respective workplace-adapted MBSR courses. It was made clear to the MBSR teachers that the recommended respondents had to represent both individuals who expressed a high degree of engagement during the workplace-adapted MBSR course as well as individuals with lower levels of engagement. In total, 76 respondents representing the four participating companies were included in a pre- and/or a post-intervention focus group interview. As shown in Table 3, across companies, a slight majority of the respondents were female.

**Table 3:** Distribution of respondents.

Company	Respondents, *n*	Managers, *n*	Employees, *n*	Females, *n* (%)
Company 1	5	1	4	4 (80.0)
Company 2	18	8	10	12 (66.6)
Company 3	25	5	20	16 (64.0)
Company 4	28	8	20	9 (32.1)
Total	76	22	54	41 (53.9)

### Data collection

Semi-structured focus group interviews were conducted by EM and EB, with EM as the primary interviewer and EB as observer and substitute. In Company 1, management consisted of one manager, therefore an individual semi-structured interview was conducted with this manager at both pre- and post-intervention. In total 14 pre-intervention focus group interviews and 13 post-intervention focus group interviews were performed between March 2020 and May 2021. At the beginning of all conducted interviews, respondents received detailed information about the study, the use of data, and the possibility to withdraw from the study at any given time. All respondents provided oral consent.

Apart from being a researcher, EM is an experienced interviewer and has in-depth knowledge of and practical experience with establishing safe sharing environments. EM is an organizational psychologist with no previous personal or professional experience of mindfulness or MBIs. EB holds an MSc in public health and has personal experience with and scientific knowledge of mindfulness and MBIs.

All 14 pre-intervention interviews were conducted before the two-hour information session using a semi-structured interview guide. The purpose of the interviews was two-fold: (1) to get insights into employees and managers knowledge of mindfulness and their patterns of behavior during stressful situations and (2) to gain insight into the social dynamics of the workplace as an organization. Therefore, the interview guide consisted of 9 themes: (a) Thoughts related to mindfulness, (b) Information and thoughts about the project, (c) Stress, (d) Coping with stress/overload, (e) The company’s prioritization of well-being, (f) Collaboration within the company, (g) Communication and tone, (h) Feedback culture within the company and (i) Expectations regarding one’s own and the organisation’s participation in the research project (themes e-i) will not be elaborated on in the present study.

The 13 post-intervention interviews were conducted following the implementation workshop. The purpose of the post-intervention interview guide was to gain insights into the intervention’s possible effects on the mental health skills of employees and managers as well as possible effects on the social dynamics within work groups and the organization as a whole. The post-intervention interview guide consisted of 8 themes: (a) Experiences of participating in the intervention, (b) Abilities to be in the present moment and notice bodily sensations, (c) Stress and behavior during stress, (d) Interpersonal relations, (e) Feedback culture and prioritization of well-being, (f) How the intervention is being narrated in the organization, (g) Facilitating and inhibiting factors for engagement and (h) Wishes for the future implementation of mindfulness. The organizational effects are to be published elsewhere and hence, this study focuses on themes a-d.

Due to the Covid-19-pandemic, most interviews were conducted live online using Zoom. Hence, 19 of the 27 interviews (70.4%) were performed live online. These 19 interviews were recorded using the record-function in Zoom. The remaining 8 interviews, performed in person, were recorded using a Dictaphone. During the interviews, EB made notes of seemingly relevant statements, demeanour and atmosphere.

### Analysis

Verbatim transcription of the interviews was conducted by EB. The transcriptions included pauses, length of pauses, changes in voice and pitch as well as changes in body language. Data analysis was performed following the four steps of inductive qualitative content analysis (Schreier, 2014; Bjerrum and Lyhne, 2021): 1. Create an overview of the data, 2. Identify and extract meaning units using analytical questions, 3. Categorize meaning units into descriptive categories, and 4. Transversal analysis of categories to condense explanatory themes.

Firstly, EB read through all transcripts and noted preliminary analytical reflections. Secondly, meaning units were identified and extracted using three analytical questions: 1. How do employees and managers describe their awareness in the present moment? 2. What reactions to stress are described by employees and managers? 3. How do employees and managers relate to themselves and their felt needs? These analytical questions were based on the programme theory developed before the trial commenced. EB and EM independently identified meaning units on parts of the transcripts. Diverging identifications were discussed until agreement was reached. EB then conducted the identification and extraction of meaning units in all transcripts. Thirdly, meaning units were categorized into descriptive categories. Four pre-intervention and six post-intervention categories were condensed. During this analytical step, notes made during the interviews and the first analytical step were consulted to ensure that the individual meaning unit was not extracted from its context, leading to potential risk of over-interpretation. Following initial categorization, inter-coder validation of the descriptive categories was performed. Inter-coder agreement was 75.8%. The majority of disagreement was due to differences of the interpretation of specific meaning units. Upon discussion and consultation with notes, agreement was reached on interpretation of all meaning units and their respective categories. All categories were found to be valid. Fourthly, transversal analysis across categories was conducted. In collaboration, EM and EB compared categories that resulted in two explanatory themes. EM and EB was in close contact and had running discussions during the analytical process. Descriptive categories and explanatory themes were discussed with LJ and LF.

## Results

The obtained results are generated on the basis of: 1. Pre-intervention focus groups, 2. Post-intervention focus groups. First, results from pre-intervention focus groups followed by those from post-intervention.

### Qualitative pre-intervention focus groups

Pre-intervention, four descriptive categories were condensed using inductive content analysis: 1. Bodily sensations and awareness in stressful situations, 2. Reactive and passive behavior during stressful situations, 3. Differences in perception as a stressor, 4. Self-criticism and low ability to practice self-care.

#### Bodily sensations and awareness during stressful situations

When asked about what bodily sensations employees and managers noticed during stressful situations, the majority were able to identify a number of these. The most commonly mentioned bodily sensations during a stressful situation were faster heartbeat, a feeling of unease, stomachache and chest tightness. Others were unable to identify any bodily sensations during stressful situations. A few of these individuals described themselves as mentally detached from their body during stress, e.g., using a metaphor of being like a machine and thereby depersonalize themselves:

“*I’m made for… I am a machine that just runs*” (Female manager, Company 3).

“*so… I do not have any signals that I’m like aware of where I think: “oh, that’s why”*” (Female manager, Company 2).

This mental detachment and lack of ability to describe bodily sensations could indicate a low level of awareness of bodily sensations, and hence lower ability to be consciously aware in the present moment, indicating lowered mental health skills.

Furthermore, some managers and employees reported having previously been on sick leave due to stress. Going through this experience, several of these individuals described becoming aware of bodily sensations during stress, which enhanced their ability to act on feelings of stress:

“*Actually, I was just on sick leave due to stress (…) if somebody has bombarded me with 10,000 questions (…), I can get a slight tingling in my fingers and at the same time, I get kind of a dry mouth and things like that*” (Female production worker, Company 3).

This indicates that previous experiences with sick leave due to stress may act as a facilitator to one’s ability to identify bodily sensations during stressful situations, posing an example of an acquired mental health skill.

#### Reactive and passive behavior during stressful situations

Across companies, employees and managers were generally able to describe specific patterns of behavior during and following stressful situations. As such, both managers and employees described examples of being reactive during stressful situations. Being reactive, respondents described acting on impulse without the ability to reflect when in a stressful situation:

“*… because I need to be able to convince myself that the decision… so, I cannot reflect on it [her reaction while in a stressful situation, red.] right in that moment. I might be able to in an hour or so*” (Female employee, Company 2).

However, a few managers gave examples of being more responsive during stressful situations, e.g., taking a break from work to get “*a birds-eye view of things*” (male manager, Company 4).

When describing specific patterns of behavior while in stressful situations, both managers and employees offered examples of specific reactions, i.e., being less patient, enhanced tendency to be defensive of oneself to others or wanting to avoid whatever is causing stress:

“*personally, I react kind of outward, I think… Or not outward, I’m not like yelling and screaming, but… I defend myself a bit*” (Female employee, Company 2).

Others spontaneously mention that they occasionally get “*passive*” and mentally shut down during stressful situations:

“*(…) then I begin to push things around without solving them. Then I notice that I get inefficient, I get passive*” (Female employee, Company 4).

The respondents’ demonstrated ability to describe patterns of behavior in stressful situations might indicate that they were consciously aware of their behavior. However, it is not clear whether they were aware of their behavior whilst going through a stressful situation or strictly retrospectively through thinking about the transpired situation.

#### Differences in perception as a stressor

This emerging category was not part of the interview guide. However, multiple managers and employees across companies spontaneously described feelings of pressure due to others (coworkers, employees, managers) having different perceptions of a specific situation than themselves. Most commonly, respondents described experiencing lowered ability to view situations from other persons’ perspective while in a stressful situation. As such, having different perspectives on things may act as an additional stressor in an already stressful situation.

“*… He could not see how far behind he actually was. (…) and to be standing there with someone who did not share my view of the situation was almost more frustrating than it was for me to just run faster*” (Female employee, Company 2).

#### Self-criticism and low ability to practice self-care

Generally, managers and employees across companies demonstrated lack of kindness towards oneself and their own felt needs. This was expressed as self-criticism in situations where they were not able to concentrate and when they made mistakes. Furthermore, a manager in Company 1 described feeling guilty when acting on her felt needs, e.g., taking a day off from work when needed:

“*I sometimes feel guilty in relation to my employees when I say “I’m not coming tomorrow” or something, and I really want that [feeling] to go away (…) but at the same time, I scold myself a bit”* (Female manager, Company 1).

One employee in Company 1 describe how she is able to retrospectively express kindness to herself following an unpleasant or stressful situation, e.g., having made a mistake at work. However, while being in the stressful situation, she is prone to self-criticism. This might indicate an ability to reflect on a specific situation without it turning into rumination generating negative thoughts.

When becoming aware of bodily sensations indicating stress, a small number of managers expressed the ability to exercise self-care by acting on their felt needs, e.g., taking time off from work. These managers also represented individuals that were able to describe bodily sensations in stressful situations. This may indicate that being aware of bodily sensations during stress enhances ones’ ability of acting on felt needs, thereby exercising self-care. However, the majority of both managers and employees across companies gave examples illustrating a lack of attention to and taking care of ones’ physical and mental health:

“*… I’ve sometimes worked all night without sleeping. Then I’ve gone to a client meeting at 9 AM next morning (…) and then I’ve gone home again to continue working. So awake for like almost 48 h without sleeping to get the job done, right?”* (Male manager, Company 3).

### Qualitative post intervention focus groups

During the analysis of the transcribed post-intervention interview data, two individuals independently reported experiencing that feelings seemed bigger or with a higher magnitude during and after the intervention than before. This resulted in enhanced worrying about the cause of why this was the case. However, none of the other participants in the respective focus groups reported having had the same experience. Furthermore, in the beginning of several post-intervention interviews, several respondents reported not having experienced any changes from participating in the intervention. Nonetheless, during the interviews, the majority of those same individuals described specific situations, where they had noticed changes, for example in their own behavior, following the intervention.

The analysis of the transcribed post-intervention interview data revealed six categories describing the perceived impact of participating in the intervention: 1. Enhanced ability to be aware in the present moment, 2. Increased acknowledgement of how others may view things differently from oneself, 3. Increased kindness to oneself and being able to practice self-care, 4. Moving from reactive to responsive behavior in stressful situations, 5. Mindfulness as an accelerator for an ongoing personal process and 6. Practicing mindfulness – setting time aside or being mindful in everyday life.

#### Enhanced ability to be aware in the present moment

An increased ability to be aware in the present moment was apparent from the use of specific examples provided by the respondents. Both managers and employees experienced being more aware of themselves as well as their surroundings, indicating a higher degree of awareness. Being more aware of themselves resulted in an enhanced ability to sense how they were feeling, which may again influence one’s actions:

“*(…) I have a better sense of how I’m feeling. I am more able to feel joy, and I’m more able to feel if I’m sad, angry… And that also means that I can… sense what I really need*” (Female manager, Company 2).

Moreover, the ability to be more aware in the present moment manifested itself by an increased ability to concentrate during work-tasks as compared to pre-intervention. The ability to notice when one is not mentally aware in the present moment allows one to purposely return to the work-task at hand. A male employee in Company 4 stated:

“*I have a tendency to have my thoughts wander off or I start doing something else and I actually think I’ve gotten better and more conscious on staying in the assignment at hand*” (Male employee, Company 4).

Furthermore, increased awareness in the present moment seemed to allow employees and managers to become aware of when they ruminated, indicating a higher degree of meta-cognition. Employees across companies described themselves being able to stop rumination and to let go of thoughts concerning situations that are outside their control. Being aware of rumination enabled respondents across companies to notice what they were thinking of and then actively chose to let go of the rumination:

“*you become aware of if you are stuck in some things (…) that I become aware of “hey, I do not have to use my energy on this. I can let it go*” (Female manager, Company 2).

Being aware in the present moment was not only restricted to the ability to notice, for example, rumination or elevated concentration. Both managers and employees gave examples of being more aware of the good things in life when talking about the expressed impact of participating in the workplace-MBI:

“*I also noticed (…) where I go running and I do not even remember much of it. Like I do not remember taking in the atmosphere sensing my surroundings and now I’m just like enjoying it. Like take it all in*” (Female employee, Company 4).

As such, the ability to be aware in the present moment seems to impact employees and managers both while performing work-related tasks as well as insights into one’s emotional state.

#### Increased awareness of how others may view things differently from oneself

Pre-intervention, a category emerged that concerned experiences of individual differences in perceptions of specific situations that seemed to act as an additional stressor during an already stressful work situation. Following the workplace-MBI, managers and employees across companies reported an increased awareness of how others might perceive social situations differently from themselves. This hightend awareness of individual differences in perceptions enabled employees and managers to be curious as to how others perceive their own behavior:

“*I try to get an idea of their view of what happened is instead of having a preconceived opinion of “yeah, that’s what I said, so of course you understood it like that”*” (Female employee, Company 4).

Moreover, this increased awareness appears to allow for reflections on interpersonal differences in how one prefers work-tasks presented. This exemplifies a way for how employees and managers approach each other and communicate, in turn, possibly affecting the way employees and managers approach each other and may reflect on the level of interpersonal understanding in the workplace:

“*That you just remember (…) “how did the other person view this [situation]?” or “how is this going to be received?.” Just take the time to either understand what the other party said or take the time to explain what needs to be done*” (Female manager, Company 2).

Furthermore, results point to the intervention influencing how participants view interpersonal differences in perceptions. At pre-intervention, some interviewees appeared to view differences in perception of specific situations as a stressor in itself and something one wanted to avoid. At post-intervention, more positive feelings towards these differences seemed to have been cultivated. This was exemplified by a greater amount of respect for and accept of how others may view things, and behave, differently from oneself:

“*I do not feel that I notice these differences MORE [than before, red.] but I have become sort of more accepting and respectful, that there are others think and do and act differently than I*″ (Male employee, Company 3).

Cultivating a greater appreciation of how others view social situations differently from oneself may reduce the added feelings of frustration and stress in such situations leading to a reduction in the total amount of stress among employees and managers.

#### Increased kindness to oneself and being able to practice self-care

Several employees and managers across companies expressed an increase of self-kindness post-intervention. Contrary to what was seen pre-intervention, participating respondents reported lower degrees of self-criticism following the workplace-MBI. This was most often exemplified by how the respondents acted when making mistakes. As with the ability to let go of rumination, employees and managers experienced capabilities in letting go of mistakes thereby reducing the amount of self-criticism:

“*(…) like when I do something wrong, it’s “oh, that’s silly, that’s kind of dumb” instead of saying “Dang, you are so stupid! That’s… you are bad at all kinds of other things, too,” right? Now I just go “oops”*” (Female employee, Company 1).

The ability to show oneself more kindness may affect how one handles work tasks. One employee describe how self-criticism can add to the feeling of “*chaos*” during stressful situations (female employee, Company 2). By reducing that added feeling of chaos, the respondent felt more able to accept the stressful situation for what it was:

“*You accept it more, that this is how it is now, instead of thinking “stop, you cannot think like that!,” and then it just becomes even more chaos*” (Female employee, Company 2).

Interview data points to that the ability to practice self-kindness is intertwined with the ability to practice self-care. Pre-intervention, only a small number of managers offered examples of self-care for example by taking time off from work. However, following the workplace-MBI, several managers and employees across all participating companies reported an increased ability to practice self-care, most often exemplified as taking breaks during the workday. Pre-intervention, a male manager reported that he sometimes worked through the night to finish a presentation for work. This same manager reported not only being aware of taking breaks but of the importance of working during the day instead of evenings and nights:

“*Well… to put in some space between them [meetings, red.], so you have time during the day, so you have time to prepare the meetings. (…) That, I’m completely convinced mindfulness did to me. (…) That you need this break now and then*” (Male manager, Company 3).

However, the ability to practice self-care did not limit itself to taking more breaks during the workday. Employees experienced increased awareness of their personal boundaries and situations where these boundaries were crossed. The same individuals reported a greater ability to speak up or ask for help when they had reached their emotional boundaries:

“*But [I’ve, red.] become better at holding back and saying “I cannot make it. I’m not super-human” (…).*” *Then you [manager, red.] have to tell me what to do (…).” Not long ago, I would have just said, “Okay, I’ll keep going, and then I’ll stay a couple of hours*” (Female employee, Company 3).

#### Moving from reactive to responsive behavior in stressful situations

Pre-intervention, the majority of employees and managers showed patterns of being reactive by acting on impulse in stressful situations. Following participation in the workplace-MBI, an elevated level of responsiveness had replaced this reactiveness. As such, employees and managers described having acquired the ability to “*count to ten and breath*” in stressful situations (female manager, Company 2). The ability to respond to others instead of acting on impulse, allows the possibility of consciously choosing how to behave in situations of conflict or differences in opinion. This ability transcended into both working relationships and personal relationships. One employee in Company 1 reported that her daughter commented on her increased ability to listen and be more tolerant. At the workplace, this ability may have a positive effect on the collaboration and communication between departments, as illustrated by the below comment:

“*(…) breathe and count to ten and think, “OK, how do I want to challenge this down in the Planning department? (…)” as opposed to previously, where I would maybe just’ve said “where are those Products?!”*” (Male manager, Company 3).

However, being more aware of one’s reaction patterns in stressful situations does not necessarily eliminate reactive behavior during these situations. Sometimes, the enhanced level of awareness – in this case, the ability to notice how one reacts in stressful situations – enabled employees to notice their behavior, yet they felt unable to change this behavior while being in the situation:

“*(…) then I cannot do anything. I could see it from the outside, (…) that “now, it [reaction] has happened, now it has happened… now, my body has already reacted (…). Then I cannot do anything about it, other than noticing that it’s happening*” (Male employee, Company 4).

Furthermore, enhanced bodily awareness seemed to aid some employees in stressful situations. By using the body as an anchor to the present moment, these employees were able to handle these situations with a calmer demeanour than before:

“*[I’ve, red.] gotten better at noticing,” OK, now I need to do one thing at a time” (…) when you are at work and stressing around. Also [MBSR-teacher, red.] said to notice one’s feet if you were stressed out. Think like “now, I’ve got my foot down here. Think of that and get an overview*” (Female employee, Company 2).

Hence, participation in this workplace-MBI appears to translate into concrete changes in the employees’ and managers’ behavior in stressful situations. This affected both personal experiences of being in stressful situations and work relationships. Moreover, personal relationships may also be impacted following participation in this workplace MBI. However, only a limited number of respondents gave examples of this.

#### Mindfulness as an accelerator for ongoing personal development

This category emerged from experiences described by employees and managers who had previous experience with mindfulness practice and/or previous experiences of working on changing their behavioral patterns, e.g., by seeing a psychologist. Participating in the workplace-MBI appeared to fuel the ongoing personal development of these individuals, causing it to accelerate:

“*(…) I actually think it’s two things [ongoing personal work on changing behavior while frustrated and workplace-MBI, red.] that has been put into motion simultaneously, which then really has had a self-perpetuating effect*” (Male manager, Company 3).

Data do not give insights into potential mechanisms for this accelerating effect. However, the use of the workplace setting for practicing mindfulness may make it easier for employees and managers with a preexisting mindfulness practice to implement mindfulness into their daily lives:

“*it [mindfulness exercises] was kind of the same as what I had already started. But (…) yeah, some of it has become kind of more related to reality*” (Male employee, Company 4).

#### Practicing mindfulness – Setting time aside or being mindful in everyday life

The majority of employees and managers across companies did not establish a structured, formal mindfulness practice following participation in the workplace-MBI. However, informal exercises such as focusing on specific parts of the body when stressed were frequently employed. Moreover, following the workplace-MBI, employees and managers were aware of specific mindfulness exercises, they could use when needed:

“*(…) I’ve discovered a lot of… eh… like exercises and things like that, and I could imagine that in the future I might do a body scan just because I felt like it*” (Female employee, Company 2).

Most employees and managers perceived mindfulness exercises as means to reduce stress and not as a way of preventing it. However, several employees and managers with previous experience of practicing mindfulness, re-established or intensified their practice following the workplace-MBI:

“*(…) this course has… I already use it [mindfulness], but it has made me completely aware that I need to remember and use it more. (…) and I practice yoga about every other day, and I use mindfulness to, as you say, thoughts and living in the moment (…)*” (Female employee, Company 3).

Furthermore, following the workplace-MBI some employees and managers used mindfulness exercises in relation to physical workouts. In this way, they had implemented mindfulness in their daily lives. This speaks to three different approaches to implementing mindfulness into one’s life. Either by establishing a structured, formal mindfulness practice, or by using mindfulness exercises sporadically to diminish feelings of stress when this occurs, or by using mindfulness informally by means of being more aware in the present moment.

## Discussion

The purpose of this study was to investigate how participation in a workplace-MBI including a workplace-adapted MBSR course impact employees’ and managers’ mental health skills. A minority of respondents reported having experienced no impact from participating in the workplace-MBI intervention or adverse effects in the form of enhanced worrying of experienced feelings. However, the transversal analysis (Bjerrum and Lyhne, 2021) of the condensed categories resulted in two explanatory themes of how this workplace-MBI impacted the majority of employees and managers: “Enhanced awareness as a facilitator of kindness towards oneself and felt needs” and “Enhanced awareness as a facilitator of behavior change during stressful situations.” These two themes offer explanations as to how participating in a workplace-MBI including a workplace-adapted live online MBSR course impacts mental health skills of employees and managers. Being aware in the present moment allows employees and managers to notice how they are feeling in specific situations giving rise to both greater abilities to register individual needs and to act on these. Furthermore, the increased awareness of bodily sensations during stress and automatic reaction patterns allows employees and managers to utilize the space between stimuli and response for reflection, leading to less reactively in stressful situations.

### Enhanced awareness as a facilitator of kindness towards oneself and felt needs

Pre-intervention, the majority of the employees and managers reported a lack of kindness toward themselves, resulting in self-criticism and not responding to felt needs, for example needing a break from work. However, at post-intervention, both employees and managers across companies reported enhanced kindness toward oneself and enlarged ability to practice self-care as illustrated by, for example taking breaks during the workday. The transversal analysis showed a link between increased awareness in the present moment and the ability to practice self-kindness and self-care. Being more aware in the present moment, enabled employees and managers to notice when they were being self-critical and instead, actively treat oneself with kindness. Furthermore, the ability to become aware of thoughts was evident in categories concerning both awareness and self-kindness. Hence, by being more aware in the present moment, employees and managers expressed a greater ability to let go of both rumination and mistakes, compared to pre-intervention-categories. Previous research has found similar results of a workplace-MBI for palliative care teams, i.e., showing increased abilities to notice and to let go of ruminative thoughts (Orellana-Rios et al., 2017). Moreover, in a study on a workplace-MBI for managers, Vonderlin et al. (2021) demonstrated elevated self-care and lowered mental distress (Vonderlin et al., 2021). However, the effect on mental distress was mediated by the amount of practice performed by managers (Vonderlin et al., 2021). In the present study, only a modest number of the respondent established a formal mindfulness practice following the workplace-MBI. As such, the question is how much employees’ and managers’ mental health skills may be increased through participation in this workplace-MBI. Still, respondents reported performing informal or sporadic mindfulness practices, indicating that some respondents were engaging in mindfulness practices, despite not having established a formal mindfulness practice.

Dahl et al. (2020) propose a framework for cultivating well-being through training (Dahl et al., 2020). This framework describes four dimensions relating to strengthening well-being through mental training: *awareness*, *connection*, *insight*, and *purpose* (Dahl et al., 2020). Training one’s abilities within these dimensions are linked to skills that promote well-being (Dahl et al., 2020). Following participation in this workplace-MBI including a workplace-adapted MBSR course, employees and managers expressed enhanced capabilities regarding both *awareness* and *insight*. According to the framework, awareness relates to a more attentive way of perceiving both external and internal cues, i.e., bodily sensations (Dahl et al., 2020). Previous research has found that not being aware in what you are doing in the present moment is associated with lower levels of perceived happiness (Killingsworth and Gilbert, 2010). Hence, enhancing awareness in the present moment may result in increased perceived happiness. Dahl et al. understand insight as self-knowledge of how one relates to for example, emotions and thoughts as well as one’s “*sense of self*” (Dahl et al., 2020). By enhancing awareness and insight, some employees and managers can approach themselves with newfound kindness. Previous research has underpinned the importance of self-awareness in order to become more self-kind and thereby less self-critical (Barnard and Curry, 2011). Self-criticism has previously been found associated with major depressive disorder (MDD), indicating that self-criticism may increase the risk of developing MDD (Ehret et al., 2015). Furthermore, treating oneself with compassion has been found to be associated with lower levels of depression and anxiety (Ehret et al., 2015; Muris et al., 2016) and enhanced well-being (Barnard and Curry, 2011). Therefore, the skill of self-kindness may serve as a protective mental health skill. Thus, actively training the two dimensions *awareness* and *insight* through a workplace-MBI including workplace-adapted MBSR, can impact the mental health skills of employees and managers. Furthermore, the results of the present study indicate that utilizing workplaces as settings for MBIs may help integrate mindfulness practices into the way, employees and managers conduct their work. This emphasises the potential for using workplaces as mental health promoting and preventive settings.

### Enhanced awareness as a facilitator of behavior change during stressful situations

The transversal analysis demonstrated a link between employees and managers’ expressed enhanced awareness in the present moment and their ability to behave more responsively in stressful situations. Hence, by being more aware, employees and managers were sometimes able to utilize the space between stimuli and reaction to choose a more responsive approach to for example, a disagreement between colleagues. In a study among adolescents, Zimmer-Gembeck et al. (2021) found that dispositional mindfulness in the form of mindful awareness directly affected involuntary emotional reactions to stress resulting from peer relationship problems, such as conflicts (Zimmer-Gembeck et al., 2021). Moreover, mindful non-reactivity was found to have a direct effect on the experience of fewer involuntary reactions to stress (Zimmer-Gembeck et al., 2021). These effects appear to be mirrored in the responses by employees and managers in the four included companies in the present study. The expressed changes from reactive to responsive behavior during stressful situations relates directly to WHO’s definition of mental health: “… *can cope with the normal stresses of life…”*(WHO, 2018b). Hence, these expressed changes in behavior in stressful situations may indirectly affect the mental health of employees and managers *via* enhanced mental health skills. In a study of how an MBI impacted university employees, respondents stated that following participation in the MBI, they listen more actively, think and then respond during interactions at work (Hegney et al., 2021). However, as pointed to in the present study, increased awareness may not always lead to changes in the automated patterns of reaction in stressful situations. This might reflect that there is a leap from awareness to actual change, or that there is a need to practice over a longer period for the increased awareness to result in actual change.

Pre-intervention, differences in how people perceive social situations could cause additional stress in stressful situations. Employees and managers expressed that being aware of differences in perception in a stressful situation affected how they related to these and buffered the feeling of added stress caused by differences in perception. Changes in how one relates to interpersonal differences in perceptions may play a role in enhancing the second dimension, *connection*, of the well-being training framework by Dahl et al. (2020). Positive interpersonal relationships have previously been found to be a protective factor against for example, depression (Santini et al., 2015). Additionally, previous research in workplaces has demonstrated associations between the quality of social relationships and mental health (Rydstedt et al., 2012). As expressed by one employee, changing how one relates to differences in perception may generate a greater amount of respect for other people’s views. The enhanced respect might affect how one interacts with others and hence the social relationships. Thus, these changes in how employees and managers manage emotions and their relations to others serve as mental health skills, which may in turn impact their mental health.

### Strengths and limitations

One of main strengths of this study is that it utilized a population-based strategy, thereby making this mental health promoting and preventive intervention available for all employees and managers within the participating companies. Furthermore, the interview guide was based on theoretical assumptions from previous research and the programme theory (Supplementary material). Adding to the strength of the present study is that the delivered workplace-MBI was systematically developed in accordance with the recommendations on adaption of MBIs (Crane et al., 2017) by experienced MBSR teachers. Moreover, the study included a large number of respondents from four private SMEs representing four different business areas. The similarities in responses from employees and managers across companies imply that these results may represent implications one could expect to find in private SMEs across business areas. Moreover, given that EM did not have prior professional or personal experience of mindfulness or MBIs this contributes to the internal validity of the study, ensuring the results are not a product of preunderstanding.

Nonetheless, the study has some limitations. Being a part of a research project with an overall aim of creating healthy work environments, data for the present study was collected using focus group interviews. By being collected in focus groups, data may not offer the same insights to respondents’ lived experiences, as individual interviews (Halkier, 2010). Therefore, these results are situated in social contexts and may be influenced by group dynamics between the focus group participants. However, as demonstrated in the explanatory themes, similar results have been found among individuals in previous studies (Orellana-Rios et al., 2017; Hegney et al., 2021), indicating validity of the obtained results. An added benefit of conducting data collection using focus groups was that respondents validated the experiences of peers and followed up with their own examples of similar experiences. However, during the focus groups, sometimes employees and managers expressed disagreement or did not recognize experiences from peers. This tendency may indicate that respondents generally felt safe sharing personal experiences relating to the intervention in the focus group setting. Future research should include individual interviews to obtain data on the lived experiences of the respondents. Another limitation was that the majority of data was collected live online *via* Zoom. To the extent that respondents were not present in the same room, this format challenged the interpretation of group dynamic and body language. This may have affected how the respondents participated in the discussions within the focus groups. However, in the majority of focus groups, EM and EB did not experience that the online format hampered the participation of the respondents. Furthermore, EM and EB made sure to invite all respondents into the discussions within the respective focus groups.

### Conclusion

This study sheds light on how the mental health skills of employees and managers in private SMEs in Denmark may be impacted by a workplace-MBI including a systematically developed online workplace-adapted MBSR course. Following participation in this workplace-MBI, employees and managers expressed enhanced abilities in being aware in the present moment by for example, noticing how one is feeling and noticing when ruminating. Furthermore, participants demonstrated more kindness towards themselves and were more aware of how they behaved during stressful situation. The enhanced awareness facilitated changes in their abilities to practice self-kindness and self-care and to change behavior during stressful situations from reactive to responsive. These results indicate a strengthening of the mental health skills of employees and managers participating in this workplace-adapted MBI. Data underlying these results are based on focus group interviews. Future research should include individual interview to gain insights into the lived experiences of respondents. This may contribute to more in-depth knowledge of how individual employees and managers experiences of participating in the intervention and its potential impact on their mental health skills.

## Data availability statement

The raw data supporting the conclusions of this article will be made available by the authors, without undue reservation.

## Ethics statement

Ethical review and approval was not required for the study on human participants in accordance with the local legislation and institutional requirements. Written informed consent for participation was not required for this study in accordance with the national legislation and the institutional requirements.

## Author contributions

LJ and EM designed the study. EM and EB developed the interview guide and collected data and performed inter-coder reliability tests of the validity of the coding and categorization, were in dialog throughout the analysis process, and finally discussed the results with LJ and LF. EB transcribed the interviews, performed coding, and categorization of all interviews, and drafted the manuscript. LJ, EM, and LF contributed with valuable corrections. All authors contributed to and approved of the final submitted manuscript.

## Funding

This study was funded by The Velliv Association. Grant number: 19-0506.

## Conflict of interest

The Danish Center for Mindfulness, Aarhus University, offers MBSR courses and MBSR teacher training. The Danish Center for Mindfulness receive payment for both services. The authors declare no conflict of interest.

## Publisher’s note

All claims expressed in this article are solely those of the authors and do not necessarily represent those of their affiliated organizations, or those of the publisher, the editors and the reviewers. Any product that may be evaluated in this article, or claim that may be made by its manufacturer, is not guaranteed or endorsed by the publisher.
