# Supplementary material for: Impacting employees’ and managers’ mental health skills using a workplace-adapted mindfulness-based intervention
Source: Front Psychol. 2022 Dec 6;13:1020454. doi: 10.3389/fpsyg.2022.1020454 (PMC9763721; doi:10.3389/fpsyg.2022.1020454)
Supplement: Supplementary file 1 [file Table_1.DOCX]

Supplementary Material

**1. Programme theory of the intervention**


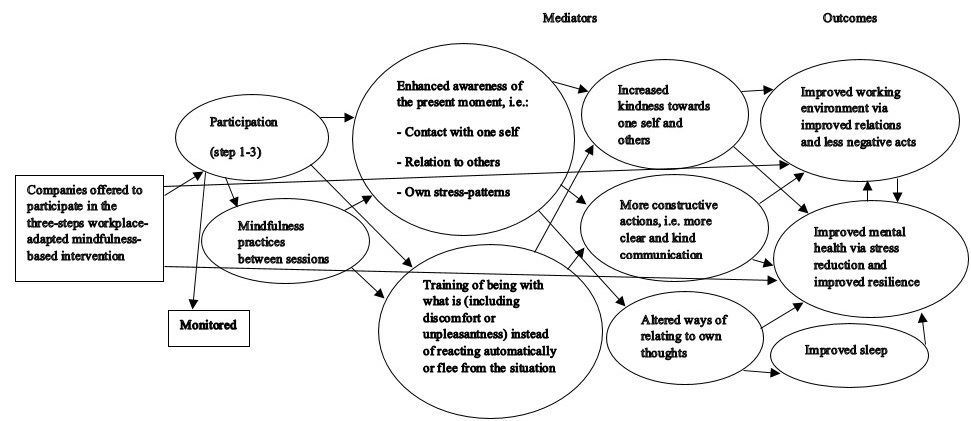


Supplementary figure 1: Programme theory of the three-step workplace-adapted mindfulness-based intervention including a 10 weeks live online workplace-adapted mindfulness-based stress reduction course.
